# Optimization of Fixations for Additively Manufactured Cranial Implants: Insights from Finite Element Analysis

**DOI:** 10.3390/biomimetics8060498

**Published:** 2023-10-19

**Authors:** Fariha Haque, Anthony F. Luscher, Kerry-Ann S. Mitchell, Alok Sutradhar

**Affiliations:** 1 Department of Mechanical and Aerospace Engineering, The Ohio State University, Columbus, OH 43210, USA; haque.73@osu.edu (F.H.); luscher.3@osu.edu (A.F.L.); 2 Department of Plastic Surgery, The Ohio State University Wexner Medical Center, Columbus, OH 43210, USA; mitchell.2079@osu.edu

**Keywords:** cranial implants, fixation points, patient-specific design, finite element analysis

## Abstract

With the emergence of additive manufacturing technology, patient-specific cranial implants using 3D printing have massively influenced the field. These implants offer improved surgical outcomes and aesthetic preservation. However, as additive manufacturing in cranial implants is still emerging, ongoing research is investigating their reliability and sustainability. The long-term biomechanical performance of these implants is critically influenced by factors such as implant material, anticipated loads, implant-skull interface geometry, and structural constraints, among others. The efficacy of cranial implants involves an intricate interplay of these factors, with fixation playing a pivotal role. This study addresses two critical concerns: determining the ideal number of fixation points for cranial implants and the optimal curvilinear distance between those points, thereby establishing a minimum threshold. Employing finite element analysis, the research incorporates variables such as implant shapes, sizes, materials, the number of fixation points, and their relative positions. The study reveals that the optimal number of fixation points ranges from four to five, accounting for defect size and shape. Moreover, the optimal curvilinear distance between two screws is approximately 40 mm for smaller implants and 60 mm for larger implants. Optimal fixation placement away from the center mitigates higher deflection due to overhangs. Notably, a symmetric screw orientation reduces deflection, enhancing implant stability. The findings offer crucial insights into optimizing fixation strategies for cranial implants, thereby aiding surgical decision-making guidelines.

## 1. Introduction

Utilizing additive manufacturing (AM), commonly known as 3D printing, in designing patient-specific implants is now gaining traction, offering a promising alternative to conventional manufacturing techniques and methods [1,2,3,4]. Integrating AM with robust CAD (computer-aided design) models of the cranial structures provides a unique opportunity to design intricate shapes, ensuring the desired form and enhanced functionality. This new technology supports the potential to construct cranial implants with desired mechanical and biological characteristics, ensuring an optimized design with minimal stress and deformation. For a cranial implant to be considered optimal, it needs to exhibit several essential biomechanical features. The foremost requirement is that it must be custom-made for the patient, ensuring a snug fit without gaps between the implant and the skull. Additionally, the implant material should mimic the gradation of the surrounding bone to reduce the chances of stress shielding. The criteria for ideal implant fixation are multi-faceted. It should provide firm fixation to prevent any movement of the implant, maintain a low profile, be biocompatible for bone integration, be easy to install and remove, be compatible with imaging systems such as MRI and CT scans, and remain cost-effective. Optimal fixation points should be strategically incorporated to promote stability while minimizing stress concentrations and deflection under external loads.

While bone grafts have stood as the gold standard for cranial defect reconstruction, the paradigm is shifting with the recent technological advancements in additive manufacturing. Novel materials such as titanium alloy (Ti6Al4V), polyether ether ketone (PEEK), and polymethyl methacrylate (PMMA) have emerged as viable contenders [5]. The increasing adoption of these materials is strengthened by their ability to produce patient-specific implants that offer commendable mechanical properties. Finite element analyses for the mechanical characterization of implants for PMMA, PEEK, and Ti6Al4V materials were conducted in [6,7,8], who reported that Ti6Al4V exhibited better resistance to deformation compared to PMMA and PEEK. Moreover, in terms of shock absorption, the Ti6Al4V implant showed a superior response compared to PMMA [8]. Yet, it is essential to consider that Ti6Al4V has a markedly higher stiffness of 110,000 MPa [9], which is approximately sevenfold than that of the stiffness of the surrounding bone, recorded at 15,000 MPa [10]. This mismatch in elastic modulus and the relative density between the titanium alloy and the surrounding bone can cause a significant stress shielding effect [11]. Such an effect has the potential to lead to gradual weakening of the skull as the implant absorbs the bulk of the load. Thus, stress shielding and the subsequent loosening of fixation devices are likely major contributors to implant failures that require revision surgeries.

The strategic design of fixation devices is vital to ensure seamless integration with the skull. This optimizes mechanical stability and mitigates the risk of infections, specifically those triggered by biofilm formation [12,13]. The interplay between implants’ geometric and mechanical properties and their implications on the loading conditions, associated stress profiles, and deformation patterns of the implant were evaluated in [14]. The combined impact of different properties was introduced by defining an assessment factor (AF), a metric that can help determine the optimal strategy based on the quantity and spatial distribution of the fixations for specific clinical cases [14,15]. The clinical success of cranial implants depends substantially on the biomechanical performance of the implant-fixation device. Additively manufactured implants can have undesired gaps due to mismatch stemming from the conversion of DICOM data to STL data [16] or from the additive manufacturing process [17] itself. Beyond serving as an attaching mechanism between the skull and the implant, the fixation devices are also crucial for gap minimization under external loads.

During an external impact, the load transfer between the implant and the skull must occur through the skull-implant interface to ensure optimal load distribution while minimizing the occurrence of local stress concentrations in the screws and plates [15]. This can be a significant concern in the fixation devices, which are typically an assembly of titanium mini-plates and micro-screws [6]. Improved stress dispersion from the implant to the skull is observed as stress and deformation diminish with an increased number of fixation points [2]. Geometric discontinuities such as holes and notches lead to localized stress concentration. Therefore, in the case of impact loading on cranial implants, continuous distribution of screws is considered the least favorable scenario [18]. Moreover, over time there is an increased risk of osteosynthesis screw-loosening. An angular fixation technique has been proposed where the skull is pulled into the implant to alleviate the screw-loosening and ensure better attachment [19]. Nevertheless, such a fixation technique comes with the risk of developing elevated strains [20]. To mitigate such strains, overlapping margins between the implant and the skull can be introduced to provide maximum contact for better dispersion of the developed strain [21]. Marcian et al. reported that high stresses are developed in the threads [6,22], which corroborates the idea of potential failure of certain implant materials.

Optimal fixation strategies should ideally produce minimal stress concentration, distributing external loads uniformly to the adjacent bone. Using fewer screws than the optimal number could lead to excessive deformation and the potential for gap formation. Hence, the principal aim is to attain optimal deformation, ensuring no gaps under external loading conditions while utilizing the minimum possible number of fixation points. In order to achieve this, it is essential to determine the critical parameters that have the most dominant effect on the deformation of the implant.

**Figure 1:**
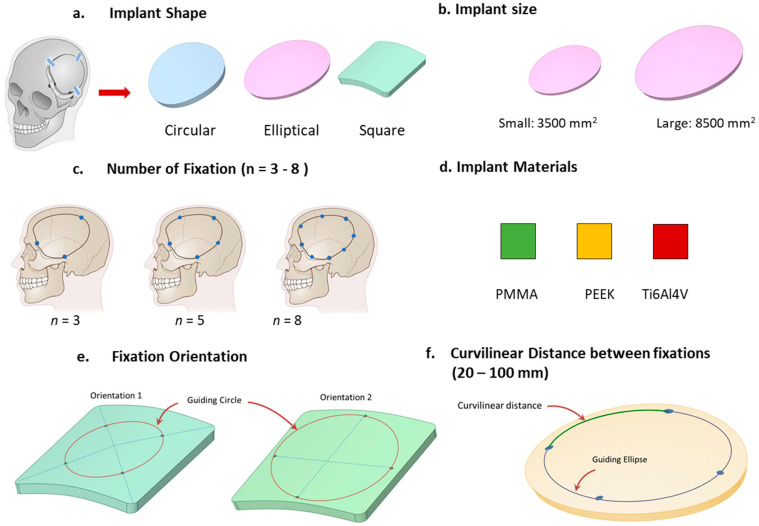
The study design, showcasing the parameters and their respective ranges, is depicted.

In this study, the deformation effects of implants in response to varying material properties, shapes, and sizes of defects/implants, external loads, and intracranial pressure (ICP) were examined. A systematic methodology was adopted to delineate the relationship between fixation and deformation, focusing on implants with diverse geometries and materials tailored for additive manufacturing. Three implant materials—PEEK, PMMA, and Ti6Al4V—were considered. A comparative analysis centered on the number of fixation screws was undertaken to ascertain the optimal count. Furthermore, geometric configurations, including circular, elliptical, and square shapes, were evaluated to understand their influence on deformation in relation to the number of screws. Figure 1 provides a comprehensive overview of the study design, enumerating the selected parameters, their classifications, and the specified ranges. The study explores the differential impacts of these parameters on the three materials and their combined influence on the efficacy of implant fixation.

## 2. Materials and Methods

### 2.1. Geometric Skull Models with Defects

A CT scan data (DICOM format) from a patient served as a foundation from which a 3D stereolithography (STL) model of a human skull was generated. The STL model was then converted to an object file (.obj) and imported into open-source software AutoDesk Meshmixer (v3.4.35; Autodesk, Inc., CA, USA) to model cranial defects. The generated defect was approximated by a convex ellipse with a major axis of 72 mm, a minor axis of 58 mm, and a perimeter of 210 mm, translating to a defect area of approximately 3480 mm^2^. Notably, the mean cranial defect size in adults spans between 2500 mm^2^ and 10,000 mm^2^ [23]. Based on these data, this study opted for two distinct defect dimensions: a ‘smaller defect’ nearing 3500 mm^2^ and a ‘larger defect’ approaching 8500 mm^2^. Three distinct geometric configurations—circular, square, and elliptical, each maintaining a consistent implant area—were examined for both defect dimensions. The assumed thickness of the skull was set at 6 mm, mirroring the average thickness observed in human skulls [24].

### 2.2. Number of Fixation Points

The study emphasizes the significance of the number of fixation points, a critical variable in implant attachment. Typically, the required holes are drilled, and fixation plates, commonly with two holes, are utilized to secure the implant to the skull. The complete skull–implant–fixation assembly was not modeled. Instead, fixations were represented as through-holes with dimensions of 2 mm in diameter and 6 mm in height, reflecting the skull’s average thickness. Clinically, at least three fixation points are used to secure the implant to the skull. This approach is often based on the principle that a triangular placement offers the highest stability [25]. Micromotions between the skull and implant gaps can potentially instigate implant failures, emphasizing the importance of ensuring tolerable deformation. Conversely, the proliferation of fixation holes and the act of drilling can induce localized stress concentrations, potentially resulting in headaches and microfractures in the skull. In order to determine the optimum number of fixation points, the deformation for skull implants are studied by incrementing the number of fixation points from 3 to 8. Most implants are fixed by drilling screws where the positioning of the fixation points is determined.

### 2.3. Orientation of the Fixation Points

**Figure 2:**
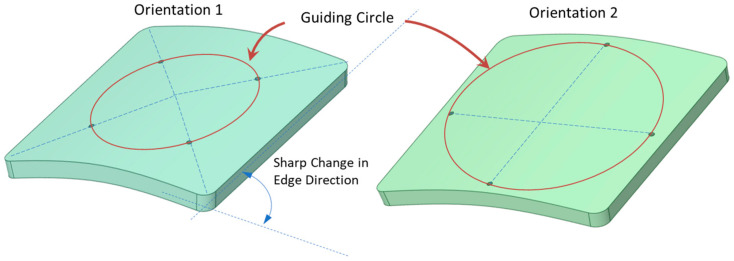
Orientation 1 depicts a fixation point positioned on the line linking the implant’s center to its corner. In orientation 2, the fixation point lies on the line joining the square’s center to its edge midpoint. The guiding circle for the placement of these fixation points, as well as the pronounced edge transition of the square implant, is illustrated.

**Figure 3:**
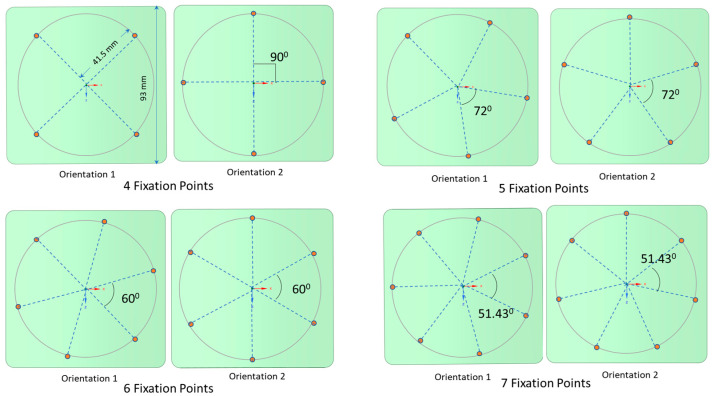
Two distinct orientations are presented for the larger square-shaped implants with four, five, six, and seven fixations. A guiding circle was created at a 10 mm offset from the edge and divided based on the number of fixations.

For enhanced stability, screws were positioned 41.5 mm away from the center of the larger square-shaped implants, spaced evenly in terms of angular distance. Two distinct orientations for the fixation points were examined, as depicted in Figure 2. In the first orientation, labeled as ‘orientation 1′, a fixation point aligns with the line connecting the implant’s center to its corner. In contrast, the ‘orientation 2′ places a fixation point along the line joining the square’s center to the midpoint of its edge. For configurations with 4, 5, 6, and 7 fixation points, both orientations were evaluated. In these configurations, the fixation points underwent rotation around a guiding circle to assess the impact of screw orientation on deformation and stress, especially in geometries with such abrupt alterations as squares (refer to Figure 3).

### 2.4. Location of the Fixation Points from the Periphery

The square-shaped implant, characterized by its four corners with an interior angle of 90 degrees, would logically suggest the need for four fixation points. For the purpose of studying this effect, only the larger square-shaped implants were considered. This choice was due to their pronounced edge direction changes, providing a valuable context for evaluating mechanical performance relative to the implant’s periphery. Four fixation points, one at each corner, were incorporated into the design.

**Figure 4:**
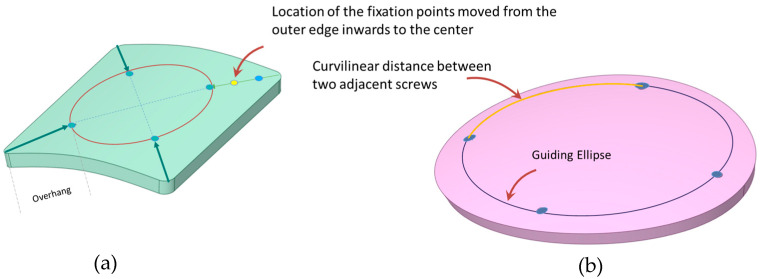
(**a**) Positioning of fixation screws is adjusted from the outer edge, moving diagonally toward the center. (**b**) Measurement of the curvilinear distance between adjacent screws using a guided circle/ellipse.

The positions of these fixation points were progressively adjusted, moving them inward from the outer edge towards the center (see Figure 4). Using a guiding circle/ellipse as reference, these fixation points were arranged to maintain equal distance from the implant’s center and consistent angular spacing between adjacent screws. The inward shifts from the outer edge were approximately set at 18 mm, 20 mm, 23 mm, 25 mm, 28 mm, and 31 mm. By relocating the fixation points more centrally, it was possible to examine how the alterations of the overhanging portion might influence the overall deformation.

### 2.5. Curvilinear Distance between Two Adjacent Screws

To understand the influence of the curvilinear distance between consecutive screws on deformation trends, implants of varying sizes and shapes, combined with different screw counts, were assessed. Given the use of geometrically shaped implants and the placement of fixation points at angular equidistance, the curvilinear distance between the screws can be determined by dividing the perimeter of the circle offset from the outer edge (which accommodates the fixation screws) by the total count of these screws (refer to Figure 4). Through this process, the optimal curvilinear distance between two neighboring screws, which offers maximum resistance to deformation, was ascertained.

### 2.6. Finite Element Analysis: Mesh Generation, Loading and Boundary Conditions, Materials

For finite element method (FEM) simulation, the skull’s geometry was imported into SpaceClaim (ANSYS Inc., Canonsburg, PA, USA) and subsequently meshed using ANSYS 2021 (ANSYS Inc., Canonsburg, PA, USA). The simulations employed 3D 20-noded solid elements. Table 1 provides a breakdown of the node and element counts for both the smaller and larger implants across varying shapes.

**Figure 5:**
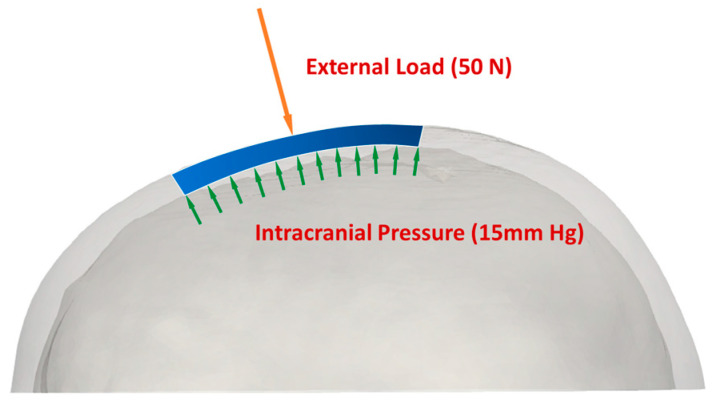
Application of loads to cranial implants: internal load represented by intracranial pressure (ICP), and external load exerted at 50 N.

The analysis focused solely on static loadings. An imposed load of 50 N, representing the approximate reactive force when resting on a level surface [15], was applied to the implant areas of 3500 mm^2^ and 8500 mm^2^. The intracranial pressure (ICP) developed within the skull balances part of this external impact (Figure 5). For adults, the average ICP usually ranges between 7–15 mm Hg [5]. An ICP value of 15 mm Hg was considered for the simulations in this study. It was assumed that the fixation holes were fixed in both the translational and rotational degrees of freedoms. Implant materials must be lightweight with sufficient strength to withstand external loading, resistant to fracture and fatigue, and be inert in response to thermal changes. Additionally, the materials should be biocompatible and nontoxic. PMMA, PEEK, and Ti6Al4V are commonly used due to their material properties and are modeled to be linearly elastic, homogenous, and isotropic in the FEM simulations. The Young’s modulus and Poisson’s ratio for these materials are listed in Table 2.

## 3. Results and Discussion

### 3.1. Effect of Number of Fixation Points on Deformation

**Figure 6:**
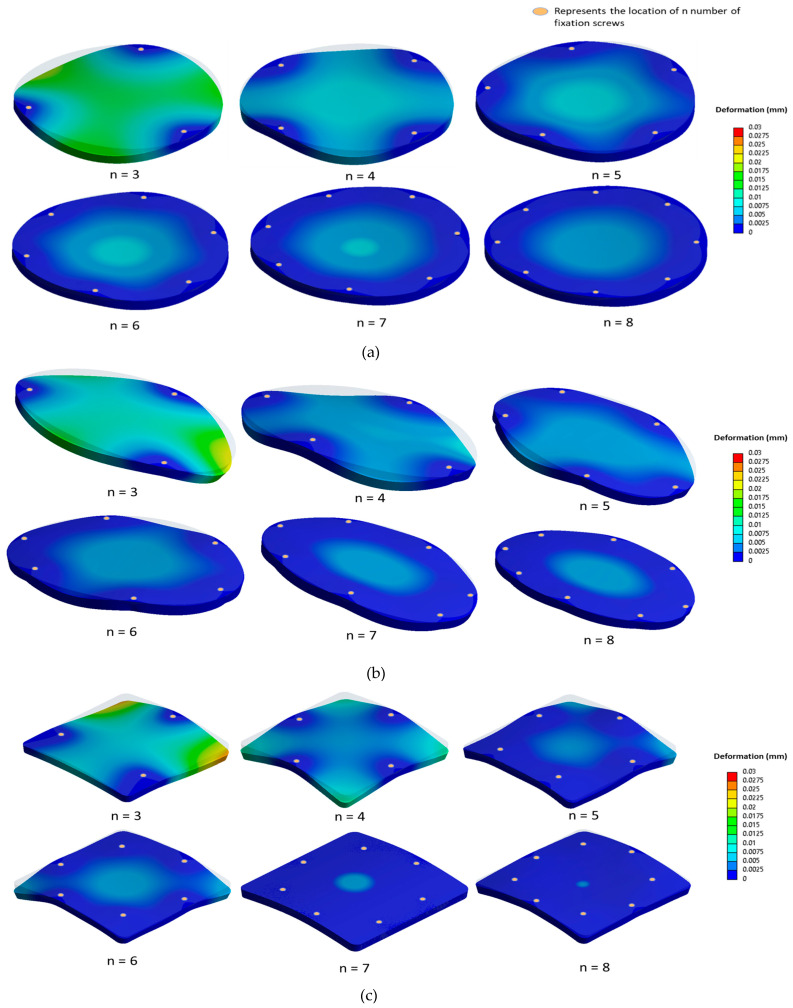
Contour plots illustrating deformation (in mm) as a function of the number of fixation screws, ranging from three to eight, for (**a**) the circular implant, (**b**) the elliptical implant, and (**c**) the square-shaped implant.

Deformation contour plots under a 50 N external load and 15 mm Hg ICP are illustrated in Figure 6 for circular, elliptical, and square-shaped PMMA implants with three to eight fixation screws. The deformation lessens as fixation points increase from three to five, reflecting the enhanced constraining of the implant edges. Beyond six to eight fixations, the periphery becomes sufficiently constrained, yielding minimal central deformation reduction.

To quantify these effects, percentage reduction in deformation, $R_{D}$, is introduced as follows:

$$R_{D}=\left(\frac{\mathrm{Deformation}\;\mathrm{at}\;n\;\mathrm{fixations}-\;\mathrm{Deformation}\;\mathrm{at}\;\left(n+1\right)\;\mathrm{fixations}}{\mathrm{Deformation}\;\mathrm{at}\;n\;\mathrm{fixations}}\right)\times 100.$$

**Figure 7:**
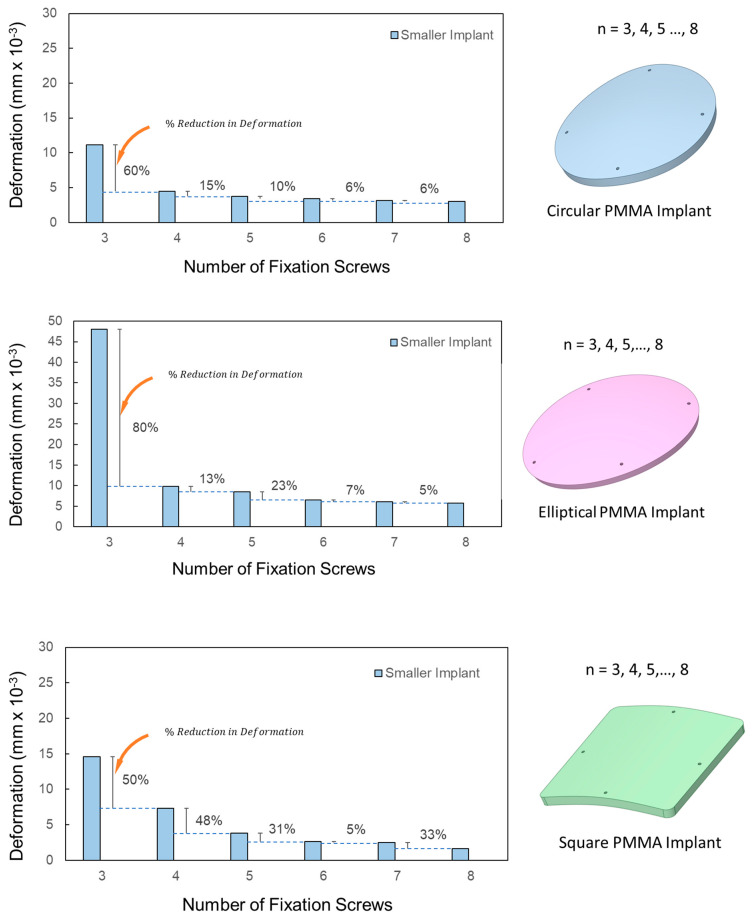
Variation in deformation with the increasing number of fixation screws in smaller PMMA implants.

The metric $R_{D}$ can help determine the optimal fixation point number for each implant. Figure 7 presents the deformation variation concerning fixation numbers for ‘smaller’ PMMA implants of different materials. For smaller defects around 3500 mm^2^, moving from three to four screws reduces deformation by 60% for circular, 80% for elliptical, and 50% for square implants. For circular and elliptical implants, deformation reduction beyond five fixations is marginal, suggesting an optimal fixation range of four–five points. This raises the question of whether this trend persists across varying defect sizes. For larger defects of 8500 mm^2^, using four screws instead of three reduces deformation by nearly 50% for circular and 75% for elliptical shapes. Square implants show approximately 50% reduction, which further drops with five to six fixations. However, the deformation reduction is under 20% for circular and elliptical types. For smaller implants with more screws, the percentage of deformation reduction dips below 10% when going from seven to eight screws for the circular and elliptical designs. Due to the square implant’s geometric symmetry and the positioning of eight screws, deformation reduces by over 30% for smaller implants and is below 10% for larger implants. Subsequent analyses for PEEK and Ti6Al4V implants of varying shapes demonstrate similar deformation trends to PMMA counterparts across shapes.

### 3.2. Effect of the Orientation of the Fixation Screws

Square-shaped implants exhibit distinct deformation characteristics in comparison to their circular and elliptical counterparts. Specifically, when the screw count in square implants is increased from four to five, there is a notable 50% decrease in deformation (see Figure 7 for square-shaped implants). This suggests that five screws might offer superior stability for square-shaped defects.

**Figure 8:**
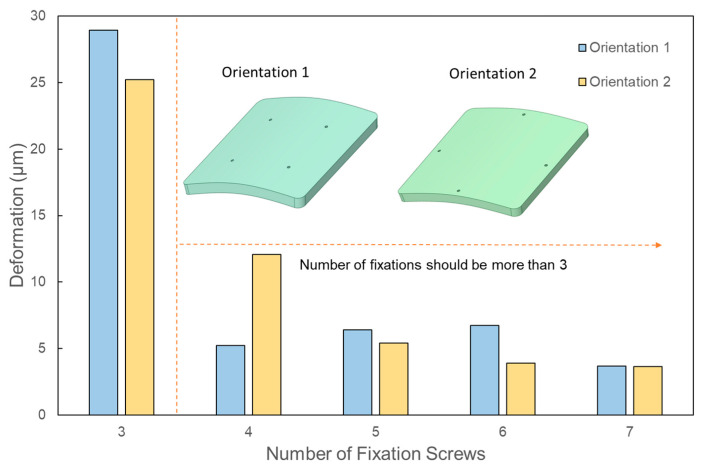
Change in the deformation corresponding to different orientations of the fixation screws for square-shaped implants.

Due to the inherent double symmetry of a square, one might assume that four or eight screws would be ideal. However, this assumption necessitates closer scrutiny, especially in terms of screw orientation. As illustrated in Figure 2 and Figure 3, two distinct screw orientations were examined across four, five, six, and seven fixation points for larger PMMA square implants. When the corners of the implant are secured, the most significant deflection transitions from the edges to the implant’s center. Interestingly, even-numbered fixations, such as four and six, displayed more noticeable variations in deformation based on screw orientation, when compared with odd-numbered fixations, such as five and seven. This distinction likely stems from the ability to apply symmetrical screw placement with even-numbered fixations in a square-shaped implant. Figure 8 details the deformations observed across the two orientations for screw counts ranging from four to seven. The deformation trends do not follow a predictable pattern across either orientation, and both orientations show almost identical deformations, with the exception of four-screw configurations.

### 3.3. Effect of the Location of the Fixation Screws from the Outer Edge

**Figure 9:**
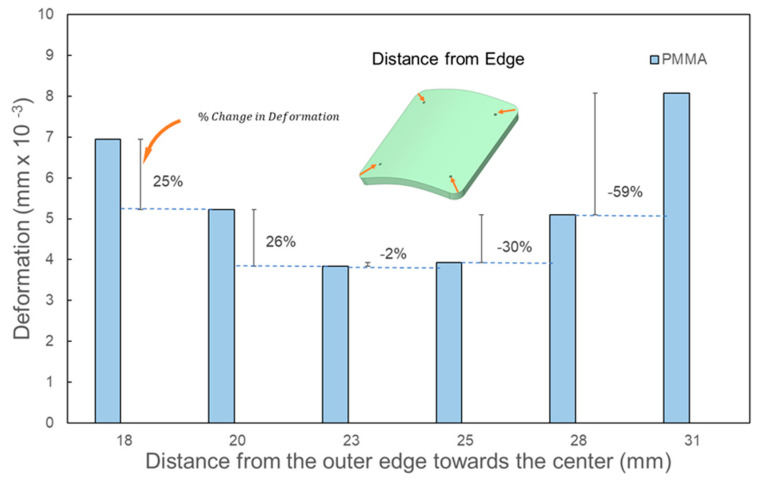
Effect of the fixation screw placement from the outer edge towards the diagonal on square implants with a large defect size (8450 mm^2^) using four screws.

Figure 6 and Figure 7 confirmed that increasing the number of screws consistently reduces the maximum deformation across all three geometric implant shapes. However, for square-shaped implants, Figure 8 demonstrated that the orientation of fixation points significantly influences deformation outcomes. Notably, when screws are anchored at the four corners of the square implants, there is a marked decrease in deformation from external loads. This observation underscores the need to further examine the effects of screw positioning relative to the edge in square-shaped implants with four fixation points. In a systematic evaluation, the screws’ placement was progressively adjusted from the implant’s perimeter towards its center along its diagonal. For all three implant materials under consideration, shifting the screw positioning further from the edge, specifically from around 18 mm to 20 mm, led to a decrease in deformation by approximately 25% under external loads, as shown in Figure 9. An additional inward shift from 20 mm to 23 mm resulted in a further 26% reduction in deformation. These findings suggest that moving screws inward consistently lessens deformation. However, this trend reverses upon moving the screws closer to the center beyond a certain point, causing deformation to spike. Specifically, a substantial 60% increase in deformation was observed when screw positioning was adjusted inwardly from 28 mm to 31 mm. This can be attributed to the overhang emerging at the square implant’s corners. Interestingly, this deformation pattern holds true for all tested implant materials, indicating that the alteration in deformation relative to screw positioning is predominantly influenced by the implant’s geometric design. It implies that screw positioning could substantially influence the peak deformation exhibited by the implant.

### 3.4. Effect of the Curvilinear Distance between Two Adjacent Screws

This analysis aims to identify the optimal curvilinear distance between consecutive screws to minimize deformation. For circular and square implants, the segment of the guiding circle’s perimeter between two neighboring screws defines the curvilinear distance. Likewise, for elliptical implants, the segment of the guiding ellipse’s perimeter (as shown in Figure 4) between consecutive screws denotes the curvilinear distance. As the number of fixation points increases, the curvilinear distance decreases proportionally, ranging from approximately 60 mm to 20 mm for the smaller implants, and from 100 mm to 30 mm for the larger ones.

**Figure 10:**
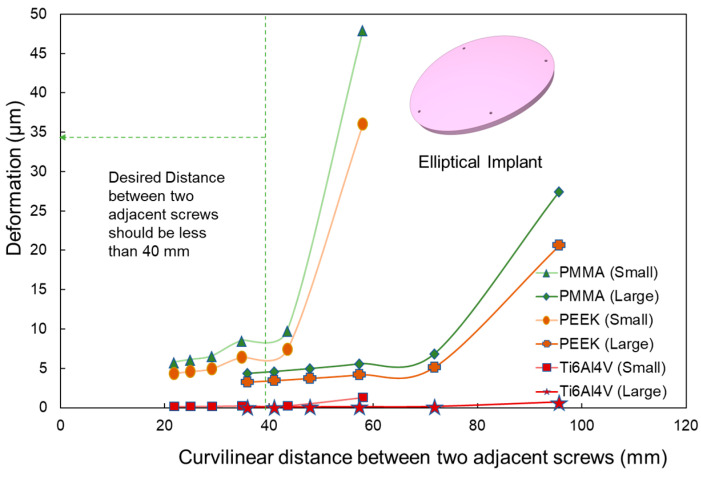
Deformation variation as a function of curvilinear distance between adjacent screws in elliptical implants. The analysis covers PMMA, PEEK, and Ti6Al4V materials for both small and large implant sizes. A notable increase in deformation is observed after a 40 mm distance.

Across all materials and geometric shapes, deformation consistently reduces as the curvilinear distance between adjacent screws diminishes, resulting in enhanced constraint. For larger defects, the deformation changes negligibly when the screw separation is below 50 mm, indicating that this range is optimal (as depicted in Figure 10). For smaller defects, the ideal separation narrows to less than 40 mm. The deformation patterns demonstrate that for the analyses conducted, distances under 40 mm yield the least deformation. However, once the curvilinear distance surpasses 55 mm, deformation begins to increase for all implant materials, sizes, and shapes. This supports prior observations emphasizing that four to five fixation points offer superior resistance to deformation in comparison with three fixation points.

### 3.5. Effect of Material Properties on Deformation

Ti6Al4V exhibits markedly lower deformation under identical external loading when compared with PMMA and PEEK. Regardless of implant size or shape, deformation in PMMA and PEEK implants is approximately 40 and 30 times higher, respectively, than in Ti6Al4V. This might suggest the superiority of Ti6Al4V due to its minimal deformation. However, Ti6Al4V’s Young’s modulus is almost sevenfold higher than bone, thereby inducing stress-shielding [11]. Furthermore, instances of screw loosening in Ti6Al4V implants in the opposite direction of the applied load have been documented [19]. Even though PMMA and PEEK show significantly higher deformation and are reported to have a higher potential for implant-related infections, the material properties of PMMA and PEEK are similar to the vicinal bone [23], effectively reducing the risk or severity of the stress shielding effect. All materials in this study are assumed to be within the linear elastic region.

## 4. Conclusions and Future Works

In this study, the impact of different variables such as implant materials, shapes, and defect sizes on the deformation of cranial implants was examined. Three different materials commonly used in additively manufactured cranial implants were investigated. Alloplastic cranioplasty, that is, skull reconstruction with these various synthetic cranial implants, is accompanied by a high risk of complications such as implant exposure, chronic pain, infections, and cosmetic deformity. Although patient factors can contribute to these complications, further studies are needed in order to delineate implant variables that may underly these issues. The evaluation focused on features of the implants that may differ based on the implant material, as well as patient-dependent variables such as defect size and shape [26,27]. Our models were based on static loading, accounting for both external loads and intracranial pressures, and they assumed the materials to be isotropic and elastic.

Our findings suggest an optimum fixation screw count of four to five, regardless of defect size. When feasible, a symmetric orientation of these fixation screws yielded superior deformation outcomes. Moreover, the study underscores the importance of maintaining a curvilinear distance under 40 mm between two screws, further solidifying the notion that a minimum of four fixations is vital for optimal deformation resistance. Intriguingly, as fixations shift inward along the diagonal, the curvilinear distance between screws decreases for the same screw count, signifying a dependence not only on the number of fixations but also on their relative position to the outer periphery.

However, our observations also indicate a threshold concerning how close the fixations can be positioned to the center; surpassing this threshold can lead to a sharp increase in deformation. This intricate interplay between the analyzed geometric parameters suggests they cannot be isolated in their effects and must be considered holistically when determining the optimal number of fixations.

Future directions for this research may incorporate machine learning approaches [28,29,30] to enhance the decision-making process on this multifaceted issue. Going forward, there is a significant opportunity to enhance the design and efficacy of cranial implants. With current efforts underway to develop point-of-care-manufactured cranial implants, with optimal implant design and fixation points determined through AI technology (in contrast to current standards of care whereby fixation points are arbitrarily assigned by the surgeon), it will become even more imperative to clarify not only implant-specific variables, but also implant behavior after fixation in the surrounding calvarium. Materials with varying properties, known as functionally graded materials (FGMs), may offer a blend of stiffness and rigidity that is ideal for implants [31]. Implants with functionally graded materials can have softer materials on the exterior, providing shock absorption, while the stiffer material in the interior has the potential to provide enhanced strength and resistance to fracture and large deformation [32]. Moreover, porous implants can provide feasible solutions [33,34]. Using novel design methods such as topology optimization can enable novel optimized implant shapes with superior performance with minimum weight [35,36,37,38,39]. This can also facilitate matching stiffness of the neighboring bone, which the authors intend to address in future studies. Additionally, the advent of additive manufacturing introduces the capability to produce hierarchical bio-lattices [40] and innovative implant structures, paving the way for implants that more accurately replicate natural bone structures.

## Figures and Tables

**Table 1:** Element and node numbers for the mesh of three different shaped implant geometries.

Geometric Size of the Defect	Geometric Shape of the Implant	Number of Nodes	Number of Elements
Smaller Defect	Circular	963,400	225,784
	Elliptical	642,633	146,988
	Square	892,892	208,611
Larger Defect	Circular	312,963	69,895
	Elliptical	304,896	68,089
	Square	298,564	66,493

**Table 2:** Material properties in the linear elastic range for PMMA, PEEK, Ti6Al4V [6].

Material	Young’s Modulus (MPa)	Poisson’s Ratio	Yield Strength (MPa)
PEEK	4000	0.38	110
PMMA	3000	0.38	65
Ti6Al4V	110,000	0.30	825

## Data Availability

Not applicable.
